# Fine-tuning signal strength in CD5 CAR-NK cells for targeted T cell cancer therapy

**DOI:** 10.3389/fimmu.2025.1674376

**Published:** 2025-09-19

**Authors:** Seona Jo, Yu Bin Lee, Seok Min Kim, Soo Yun Lee, Myeongjin Choi, Mi-lang Kyun, Seo Yule Jeong, Sunyoung Lee, Ji Hyun Kim, Yoonji Kim, Yu Jung Kim, Sora Park, Kyoung-Sik Moon, Tae-Don Kim

**Affiliations:** ^1^ Center for Gene and Cell Therapy, Korea Research Institute of Bioscience and Biotechnology (KRIBB), Daejeon, Republic of Korea; ^2^ KRIBB School of Advanced Bioconvergence, University of Science and Technology (UST), Daejeon, Republic of Korea; ^3^ Center for Global Biopharmaceutical Research, Korea Institute of Toxicology (KIT), Daejeon, Republic of Korea; ^4^ Human and Environmental Toxicology, University of Science and Technology (UST), Daejeon, Republic of Korea; ^5^ New Drug Development Center, Osong Medical Innovation Foundation, Cheongju-si, Republic of Korea; ^6^ Department of Biopharmaceutical Convergence, School of Pharmacy, Sungkyunkwan University, Suwon, Republic of Korea

**Keywords:** CD5, CAR-NK, T cell malignancies, scFv, CAR expression, on-target off-tumor toxicity

## Abstract

**Introduction:**

T cell hematological malignancies are aggressive blood cancers that remain challenging despite various treatments. Current chimeric antigen receptor (CAR)-T and natural killer (NK) therapies show potential but struggle with nonselective elimination during tumor targeting. Since CAR signal strength is determined by the single-chain variable fragment (scFv) and CAR expression levels, fine-tuning these parameters enables selective recognition of malignant cells while preserving normal cells. Here, we aimed to develop optimized CD5 CAR-NK cells (OptiCAR-NK) to achieve potent anti-tumor activity with minimized off-tumor toxicity.

**Methods:**

We engineered CD5 CAR-NK cells with different scFv and CAR expression levels. CAR expression was modulated by single-cell isolation and mRNA transfection to assess activity against both malignant and normal T cells *in vitro*. Therapeutic efficacy and safety were further validated in xenograft and humanized mouse models.

**Results:**

Optimization of scFv and CAR expression levels (OptiCAR-NK) enabled selective recognition of CD5+ malignant T cells while maintaining strong anti-tumor activity with minimal toxicity. Mechanistic analysis revealed that NK cells’ innate ability to discriminate malignant from normal T cells depends on fine-tuned CAR signal strength and endogenous ligands on target cells.

**Discussion:**

Optimized modulation of scFv and CAR expression is crucial for designing a CAR that achieves high anti-cancer efficacy and is safe in normal cells. Our results suggest a promising avenue for optimized CD5 CAR-NK cell therapy to manage T cell malignancies while minimizing off-tumor effects.

## 1 Introduction

T cell malignancies are aggressive hematologic cancers with high relapse rates and poor survival outcomes despite available treatments including chemotherapy, immunotherapy, targeted therapy, and hematopoietic stem cell transplantation (1–3). For relapsed or refractory cases, allogeneic stem cell transplantation (ASCT) remains the primary option, but its success rate is only around 30%, and many patients are ineligible (4). These limitations highlight the need for novel therapeutic strategies.

CAR-modified immune cells have shown strong efficacy against hematologic malignancies, with CAR-T therapies extensively studied in B cell cancers (5–7). However, treating malignant T cells with CAR-T cells has certain limitations due to shared antigens between normal and malignant T cells, leading to potential fratricide, T cell aplasia, and risks of malignant transformation from autologous CAR-T cells (8–10). To avoid these challenges, NK cells are a promising alternative to T cells in CAR therapy for T cell malignancies for several reasons: (i) their different phenotypes compared to T cells, prevent fratricide or contamination, (ii) their shorter lifespan reduces prolonged T cell depletion, and (iii) their innate ability to distinguish abnormal from healthy cells enhances specificity in CAR therapy (11–13). Nonetheless, when NK cells are engineered with a CAR recognizing antigens also expressed on normal cells, such as CD5, there remains a risk of on-target off-tumor effects, which requires careful CAR design.

CD5 is a therapeutic target for malignant T cells due to its high expression in these cells and certain B cell lymphomas (14, 15). While CD5 CAR-T cells can effectively target malignant T cells, they are prone to self-recognition, leading to self-elimination and reduced therapeutic efficacy (16). In contrast, NK cells do not express CD5; therefore, they prevent self-killing even after anti-CD5 CAR modification. Previous CD5 CAR-NK studies enhanced malignant T cell elimination by modifying co-stimulatory domains or introducing a nanobody-based CAR. However, both approaches have severe side effects due to the non-selective elimination of normal T cells expressing CD5 (17, 18). Therefore, achieving a balance between efficacy and safety requires careful consideration of intrinsic design factors beyond simply enhancing cytotoxicity.

CAR activity is influenced by the properties of the single-chain variable fragment (scFv), which determines antigen recognition, and by CAR expression levels, which regulate internal signaling. Variations in the sequence and structure of the scFv affect CAR expression efficiency and antigen affinity, potentially reducing off-target effects (19–23). The level of CAR expression directly correlates with internal signaling within the CAR. Previous research has predominantly focused on inducing CAR gene expression without adequately considering the intensity of CAR expression (24–28). There is a tendency towards high levels of CAR expression, showing enhanced anticancer efficacy *in vitro* and *in vivo* (29). However, excessively high CAR expression results in unfavorable clinical responses, such as cytokine release syndrome (CRS), neurotoxicity, on-target off-tumor toxicity and reduced persistence of CAR T resulting from an exhausted phenotype (30–32). To address this issue, shifting the focus from the conventional approach emphasizing CAR+ cell acquisition (33) to prioritizing CAR expression density to simultaneously attain safety and efficacy is necessary. Employing optimized scFv and CAR expression density is pivotal for developing an efficient CAR that ensures safe normal cell recognition (34, 35).

**Figure 1:**
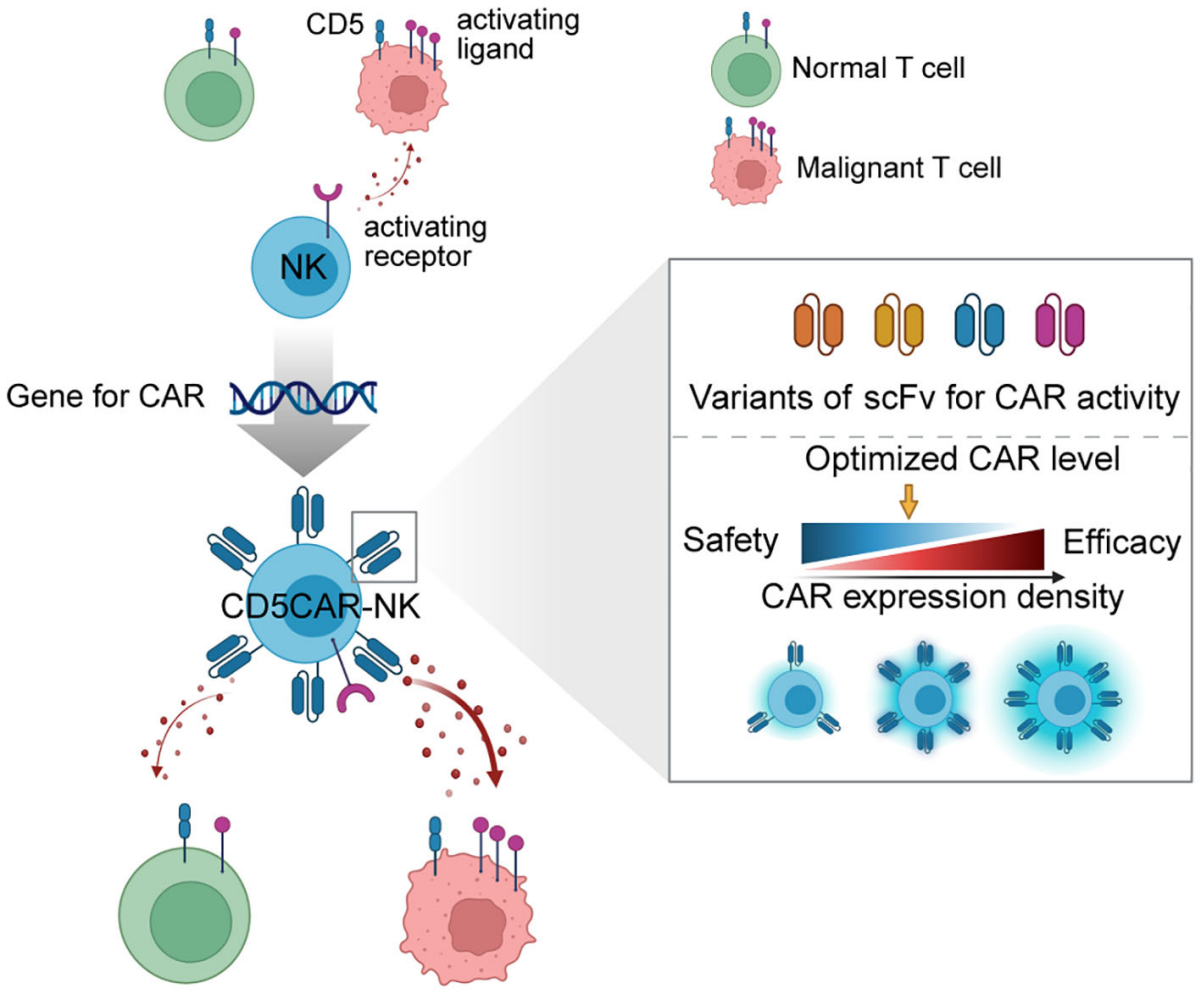
Schematic of generating optimized CD5 CAR-NK (OptiCAR-NK). This diagram illustrates the development process of CD5 CAR-NK cells, which integrates the gene for CAR expression into NK cells, creating CD5 CAR-NK with optimized properties. The right panel shows a detailed analysis of scFv variants and how adjustments to CAR expression density enable a balance between therapeutic efficacy and safety. Strategic modulation of scFv and CAR density allows for fine-tuning the CAR-NK’s ability to discriminate between malignant and normal T cells, ensuring robust anti-tumor activity while minimizing unintended targeting of healthy cells.

We investigated the effect of the characteristics of CD5 scFv and modulation of CAR density on the activity and safety of CD5 CAR-NK cells. We hypothesized that optimizing the variation of CD5 scFv and controlling their expression density would enable CD5 CAR-NK cells to selectively target and eliminate malignant T cells expressing CD5 while sparing normal T cells (**Figure 1**). Building on this hypothesis, we characterized the antigen-binding properties of CD5 scFv variants and assessed their cytotoxicity, then fine-tuned CAR expression levels using mRNA transfection and single-cell isolation. The selective activity of the optimized CAR-NK was examined against normal human immune cells and malignant T cells. Analysis of ligand expression in target cells demonstrated differential basal activity of CAR-NK cells. Through a comparative evaluation of CAR-NK cytolysis effect at various CAR levels against multiple targets, we elucidated differences in CAR-NK reactivities based on CAR levels, ligand levels, and scFv. The safety of the developed optimal CD5 CAR-NK cells (OptiCAR-NK) against human immune cells and their potential immunological risks were investigated using a humanized mouse model, and their ability to eliminate malignant T cells in immunocompromised mice was examined. Our findings suggest that OptiCAR-NK cells with modulated CAR levels and scFv effectively eliminate CD5+ T cell malignancies while minimizing adverse effects.

## 2 Materials and methods

### 2.1 Cell lines

NK92 (ATCC, CRL-2407) and CAR-NK expressing different clones (#1, #4, #11, #14) were cultured in alpha-MEM (Welgene, South Korea) supplemented with 0.2 mM inositol, 0.1 mM 2-mercaptoethanol, 0.02 mM folic acid (Sigma-Aldrich, USA), 12.5% fetal bovine serum (FBS) (R&D systems, USA), 12.5% fetal horse serum (Gibco, USA), 1% penicillin-streptomycin (PS) (Gibco, USA) and 200 IU/mL human recombinant IL-2 (PeproTech, USA). The human leukemia cell lines, MOLT4 (ATCC, CRL-1582), Jurkat (ATCC, TIB-152), CCRF-CEM (KCLB, 10119) and U937 (ATCC, CRL-1593.2), were cultured in RPMI1640 (Welgene) supplemented with 10% FBS and 1% PS. CCRF-HSB-2 (KCLB, 10120.1) and HEK293T (ATCC, CRL-3216) was cultured in DMEM (Welgene) supplemented with 10% FBS and 1% PS. Cells were incubated at 37°C and 5% CO_2_ conditions.

### 2.2 Generation of stable cell line

**Figure 2:**
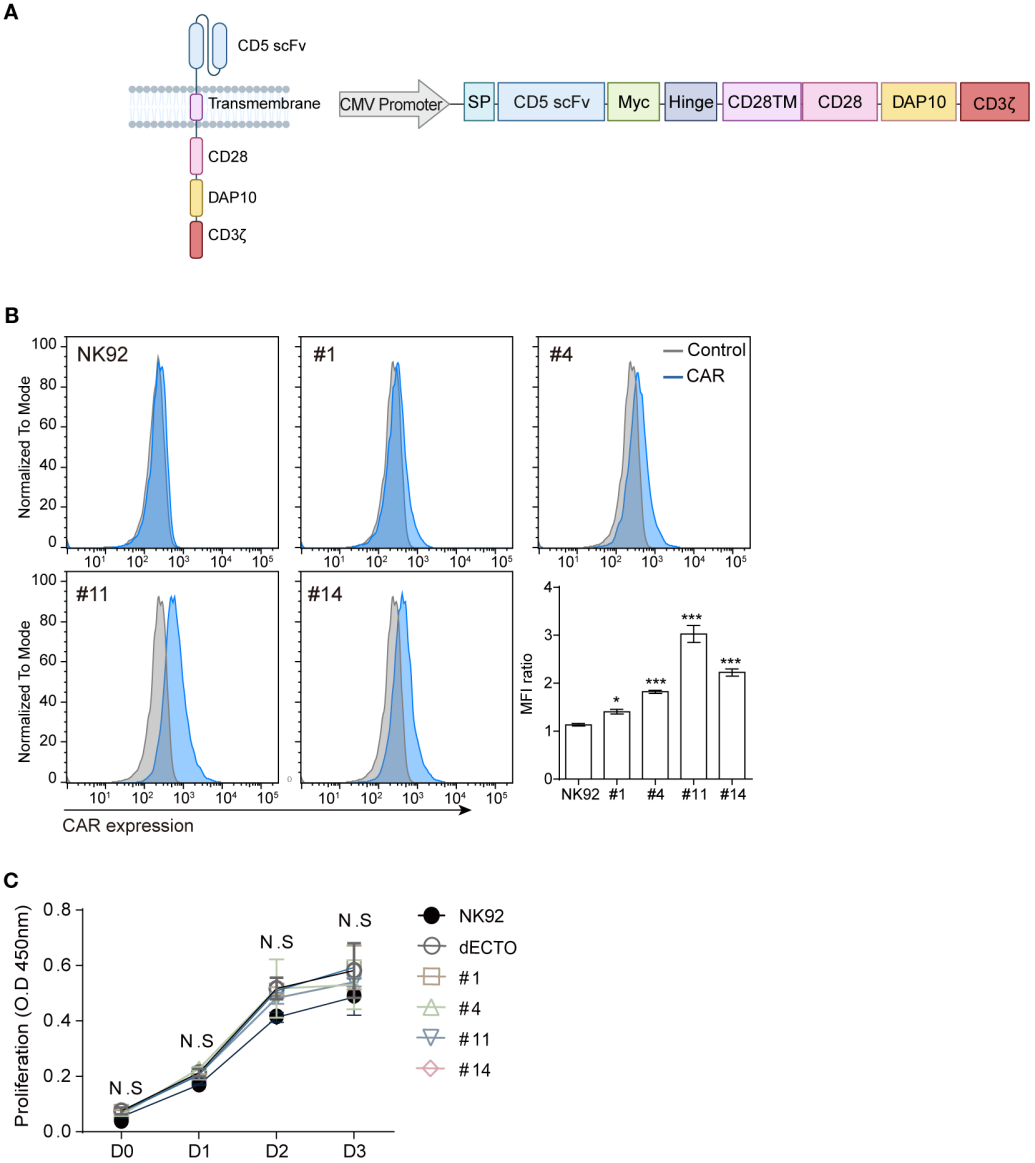
Construction of CD5 CAR and generation of CAR-NK. **(A)** Schematic representation of CD5 CAR construction: the third-generation CAR comprises CD5 scFv, myc tag, hinge, CD28 (from the transmembrane to the intracellular domain), DAP10, and CD3ζ driven by the CMV promoter. Schematic diagrams are created with BioRender.com. **(B)** Flow cytometry analysis to detect the expression levels of CD5 CAR on NK92 cells generated following an optimized protocol. Statistical significance was determined by one-way ANOVA with comparisons made relative to NK92. **(C)** Measuring the proliferative capacity of four CD5 CAR-NK cells for 3 days using the CCK8 assay. Results are from three independent experiments. Statistical significance was determined using two-way ANOVA. N.S, not significant; *p < 0.05; ***p < 0.001.

To create stably expressing CAR-NK cells, lentiviruses were produced using vector (Takara) under the control of the CMV promoter to ensure robust and consistent expression. encoding the constructs shown in **Figure 2**. Generating a lentivirus followed the previously described protocol (36). NK92 cells were transduced in complete media with titrated virus and 8 ug/ml protamine sulfate. The infected cells were selected by the optimal concentration of puromycin. Monoclonal CAR-NK cells were isolated as single cells using BD FACSAria™ Fusion Flow Cytometer and subsequently expanded.

### 2.3 Primary cell preparation

To obtain mononuclear cells (MNC) used to assess the toxicity towards normal cells of CD5 CAR-NK cells, MNC were obtained from human umbilical cord blood (UCB) using lymphoprep (StemCell Technologies, Canada) density gradient centrifugation. CD3+ cells were isolated with CD3 microbeads (#130-050-101, Miltenyi Biotec, Germany) and cultured in RPMI1640 supplemented with 10% FBS and 1% PS. To generate human primary CAR-NK cells, CD3+ T cell-depleted MNCs were isolated from UCB. It was achieved by using Rosette Sep (Stem Cell Technologies, Canada) for CD3+ T cell deletion and lymphoprep for density gradient separation. Cytokine-induced primary NK cells were differentiated from CD3- MNC with 10-6M hydrocortisone (HC), IL-15–10 ng/ml and IL-21–10 ng/ml in alpha-MEM supplied with 10% FBS and 1% PS.

### 2.4 Proliferation

Cell proliferation was assessed using Cell Counting Kit (CCK)-8 (Dojindo, Japan) according to the manufacturer’s protocol. CAR-NK cells were cultured at a density of 5 × 10^3^ cells/well in a 96-well plate and incubated for 24, 48, 72, and 96 hours in a CO_2_ incubator. Add 10ul of CCK-8 solution to each well and place in a CO_2_ incubator for 4 hours to react. The absorbance of each well was measured at 450 nm with a microplate reader (SpectraMax iD3, Molecular Devices, USA).

### 2.5 *In vitro* evaluation of cytotoxicity of CD5 CAR-NK

Cytotoxicity was evaluated by calcein-AM release assay as described previously (36). The target cells were stained with calcein-AM (#C1430, Thermo Fisher Science, USA), and co-cultured for 2 hours with effector cells according to the ratio of effector and target cells. The amount of calcein released into the supernatant is measured with a microplate reader.

The specific cytotoxicity of CAR-NK cells against on-target and off-target cells was assessed by performing Lactate Dehydrogenase (LDH) assays at specific time points (1, 2, 24, 48 hours) after co-culture. For co-culture, 1×10^5^ cells of CAR-NK and 1×10^5^ cells of either on-target (MOLT4) or off-target cells (MNC or PanT) were mixed in a total of 200 µL RPMI1640 media supplemented with 10% FBS and 1% PS and plated in a 96-well plate (n=4). For the negative control groups, set up to measure the passive release of LDH by each type of cells, 1×10^5^ cells of either CAR-NK, MOLT4, or the off-target cells were incubated individually in 200 µL of RPMI1640 media supplemented with 10% FBS and 1% PS (in a 96-well plate, n=4). For the positive control groups, established to measure the maximum release of LDH, the same conditions as the negative control group were used, but with the addition of 10% EtOH to induce complete cell death. At each time point for LDH assay, 20 µL of the working solution provided by the LDH assay kit (AB65393, Abcam, USA) was added to the 200 µL media in each well. After 1 hour of incubation, 100 µL was collected from each well, and the absorbance was measured at a wavelength of 450 nm. Cytotoxicity was calculated using the equation:


$$\begin{array}{c} \text{LDH based Cytotoxicity }\left(\%\right)=[(\text{Experimental value}–\text{Effector cell spontaneous control}–\\ \text{target cell spontaneous control})/(\text{Target cell Maximum control}–\\ \text{Target cell spontaneous control})]\times 100 \end{array}$$

To attribute LDH release specifically to target cell damage, spontaneous LDH release from effector cells alone was measured in parallel and subtracted from experimental values, ensuring that the calculated cytotoxicity reflects CAR-NK–induced target cell lysis.

### 2.6 Cytokine release assay

We conducted enzyme-linked immunosorbent assay (ELISA) to examine whether the CD5 CAR-NK cells exhibit selective changes in the secretion of inflammatory cytokines and cytotoxic granules in response to on- or off-targets. 1:1 ratio of CD5 CAR-NK cells and target cells were co-cultured for 12–24 hours. After the incubation, media were collected and centrifuged at 300 g for 10 min to obtain supernatant without cells. The supernatants were analyzed via ELISA kit [IFN-γ: K0331121, (LABISKOMA Korea), TNF-α: K0331131 (LABISKOMA), Granzyme B: ab235635 (Abcam, UK)] for the cytokines and cytotoxic granules, following the manufacturer’s protocol.

### 2.7 Flow cytometry analysis

Cells were rinsed and incubated with antibodies in FACS buffer consisting of phosphate-buffered saline (PBS) supplemented with 1% FBS and 2 mM ethylenediaminetetraacetic acid (EDTA) for 30 minutes in the absence of light at 4°C. For surface staining, the following antibody used; CD56 (#562794, BD Biosciences, USA), CD107a (#641581), CD3 (#555335), CD5 (#555352), B7-H6 (#FAB7144P, R&D Systems, USA), ULBP1 (#FAB1380P), ULBP2/5/6 (#FAB1298P), ULBP3 (#FAB1517P), MICA/B (#FAB13001P), HLA class 1 (#FAB7098P), Myc (#3739S, Cell Signaling Technology, USA), M13 (#11973-MM05T, Sino Biological, USA). To analyze three cells co-culture simultaneously, MOLT4, MNC, and CAR-NK were labeled as cell tracker Deep Red (#C34565, Invitrogen, USA), cell trace Violet (#C34571, Invitrogen), and CD56-FITC (#562794), respectively. Flow cytometric analysis was performed using a BD FACS Canto II cytometer (BD Biosciences), and the data were analyzed using FlowJo software (BD, USA). Mouse blood was obtained from the inferior vena cava and collected in K2EDTA tubes as an anticoagulant. Cells were lysed with RBC lysis buffer (BioLegend, USA) and stained with specific human antibodies CD45 (#555485, BD Biosciences). All mouse samples were analyzed using a flow cytometer (Cytoflex S, Beckman coulter, USA). Data were analyzed by CytExpert Software v2.4 (Beckman coulter, CA).

### 2.8 Transient transfection

To knock down the activating ligand B7-H6, small interfering RNA (siRNA) was delivered via electroporation into the malignant T cell lines MOLT4 and Jurkat. Target cells (1 × 10^6^) were transfected with10μM AccuTarget™ Negative Control siRNA (Bioneer, SN-1003) as a control or siRNA, which targets B7H6 (siRNA sequence: 5′-CCACAAAGUCUGAGAAACA-3′) (37) in 100 μl of Opti-MEM (Gibco). After 24 hours, the expression level of B7-H6 on target cells was analyzed using flow cytometry and cytotoxicity was assessed. mRNA-based CAR-NK was generated by synthesizing mRNA using the *in vitro* transcription kit (#AM1345, Invitrogen) with a pBluescript SK+ vector encoding the constructs illustrated in **Figure 2**. Primary NK or NK92 cells (1 × 10^6^) were transfected with different concentrations of mRNA in 100 μl Opti-MEM. After 5–7 hours, primary NK or NK92 cells were analyzed for CAR expression levels by flow cytometry following transfection and subsequently utilized for cytotoxicity assays. All electroporation was performed using a NEPA21 electroporator (Nepa gene, Japan) according to the manufacturer’s instructions.

### 2.9 Western blot

Cells were subjected to lysis using protein lysis buffer (#4719956001, Roche, Switzerland) supplemented with protease and phosphatase inhibitors (#4906837001, Roche). Lysates were prepared from CAR-NK and target cell co-cultures without prior separation, consistent with previous report (38, 39). This approach was used to assess overall activation tendency, not NK-exclusive signaling. Protein concentrations were determined using a Pierce BCA Protein Assay Kit (#23225, Thermo Scientific, USA). Cell lysates containing 10–20 μg of proteins were applied to 12% SDS-PAGE gels and transferred to 0.45 μm PVDF membrane (#IPVH00010, Merck Millipore, Germany). After the transfer, membranes were blocked with 5% skim milk for 40–60 min at room temperature and then incubated with primary antibodies overnight at 4°C. The following primary antibodies were used: GAPDH (#5174), pERK (#9101), ERK (#9102), pAKT (#9275), AKT (#9272) purchased from Cell Signaling Technology. Subsequently, the membranes were washed and exposed to horseradish peroxidase (HRP)-conjugated anti-rabbit IgG (#31460, Thermo Fisher). The membranes were developed by SuperSignal West Pico Chemiluminescent Substrate (#34078, Thermo Fisher) and western blot images were detected using WSE-6100 LuminoGraph (ATTO, USA).

### 2.10 Animals

Six-week-old females as NOD.Cg-Prkdc*^scid^* IL2γg*^tm1 Sug^*/JicKoat (NOG) mice were purchased from Koatech and allowed an acclimate period of 7 days. The mice were assigned randomly into two groups. The mice were housed in a specific pathogen-free (SPF) space under the following conditions: the temperature at 22 ± 1°C, the humidity of 55 ± 10%, and 12/12h of the light-dark cycle. All except unscheduled dead mice were included for analysis. Mice were observed daily for clinical signs and survival. All protocols were designed to minimize the number of animals and their pain or stress. In this study, the ARRIVE guidelines were applied.

### 2.11 Cancer xenograft mouse model

To create a xenograft mouse model, MOLT4 (3×10^5^) cells expressing firefly luciferase (MOLT4-Luc) were injected into the tail vein of mice. The dECTO and CAR-NK92 cells (3×10^6^) were injected intravenously through the tail vein of mice four times for 7 days after MOLT4 injection. D-Luciferin (Perkin- Elmer, USA) was administered into the abdominal cavity of mice every 3 to 4 days, and cancer cell growth was monitored using bioluminescence imaging (BLI) (IVIS Spectrum, Perkin- Elmer, USA).

### 2.12 Engraftment of human PBMC in mice

Twice intraperitoneal injection of Busulfan^®^ (Otsuka America Pharmaceutical, Japan) with 25 mg/kg into 8 female mice at least 48 hours before PBMC injection. Human peripheral blood mononuclear cells (PBMC) from a healthy donor (#CC-2702) were obtained from Lonza. Cryopreserved PBMC were defrosted in a water bath at 37°C. Cells were washed twice in PBS and resuspended in the RPMI1640 at a density of 1-2×10^6^ cells/100 μl and injected into the tail vein of each mouse. The order of treatments and measurements was recorded.

### 2.13 Ethics approval and consent to participate

Human studies were approved by the Korea Research Institute of Bioscience and Biotechnology (KRIBB) Institutional Review Board (P01-201610-31-002). Animal studies were approved by the Animal Experimental Ethics Committee of KRIBB (KRIBB-AEC-23087) and by the Institutional Animal Care and Use Committee of Korea Institute of Toxicology (IAC-22-01-0214).

### 2.14 Statistical analyses

Statistical analyses and significance were determined using GraphPad Prism v6 software (GraphPad Software Inc. LA Jolla, CA). The methods used to analyze the statistical significance of the data were thoroughly mentioned. ANOVA was used to assess overall group differences, followed by Dunnett’s multiple comparisons test to compare experimental groups with the control. Statistical significance is indicated. Error bars represent mean values ± standard deviation (SD) unless otherwise noted. The degree of significance is indicated as follows: N.S (not significant), ∗p< 0.05; ∗∗p< 0.01; and ∗∗∗p< 0.001.

## 3 Results

### 3.1 Generation of stable CD5 CAR-NK cells with different scFvs

We screened various anti-CD5 scFvs and selected the top four cells with high binding efficiency against CD5-expressing T cell leukemia cell line Jurkat (**Supplementary Figure 1A**) to obtain CD5 CAR-NK cells with enhanced efficacy against cells expressing the target antigen. Additionally, the monoclonal phage ELISA assay against CD5 (**Supplementary Figure 1B**) further validated the binding affinity of these scFvs, confirming their potential for effective targeting of CD5+ cells. Additionally, the conjugation assay against CD5+ target cells provides further insights into binding efficiency differences among scFv variants (**Supplementary Figures 1C–E**).

The four scFvs (designated as clone #1, 4, 11, and 14) were integrated into the CAR construct, where CD28 and DAP10 served as the co-stimulatory domain, and CD3ζ was the signal transduction domain (**Figure 2A**). Four types of stable CD5 CAR-NK cells were obtained by lentiviral transduction (**Figure 2B**). After lentiviral transduction, we performed a proliferation assay to compare the expansion of NK92 cells, ecto-domain deleted CAR-NK cells (dECTO) as a negative control, and four CD5 CAR-NK cell variants. The dECTO construct lacks the antigen recognition domain (scFv), preventing target binding and activation while preserving intracellular signaling. The results showed no significant differences in the expansion abilities over three days of culture (**Figure 2C**).

### 3.2 CD5 CAR-NK cells show robust anti-cancer activity against malignant T cells based on scFv type

**Figure 3:**
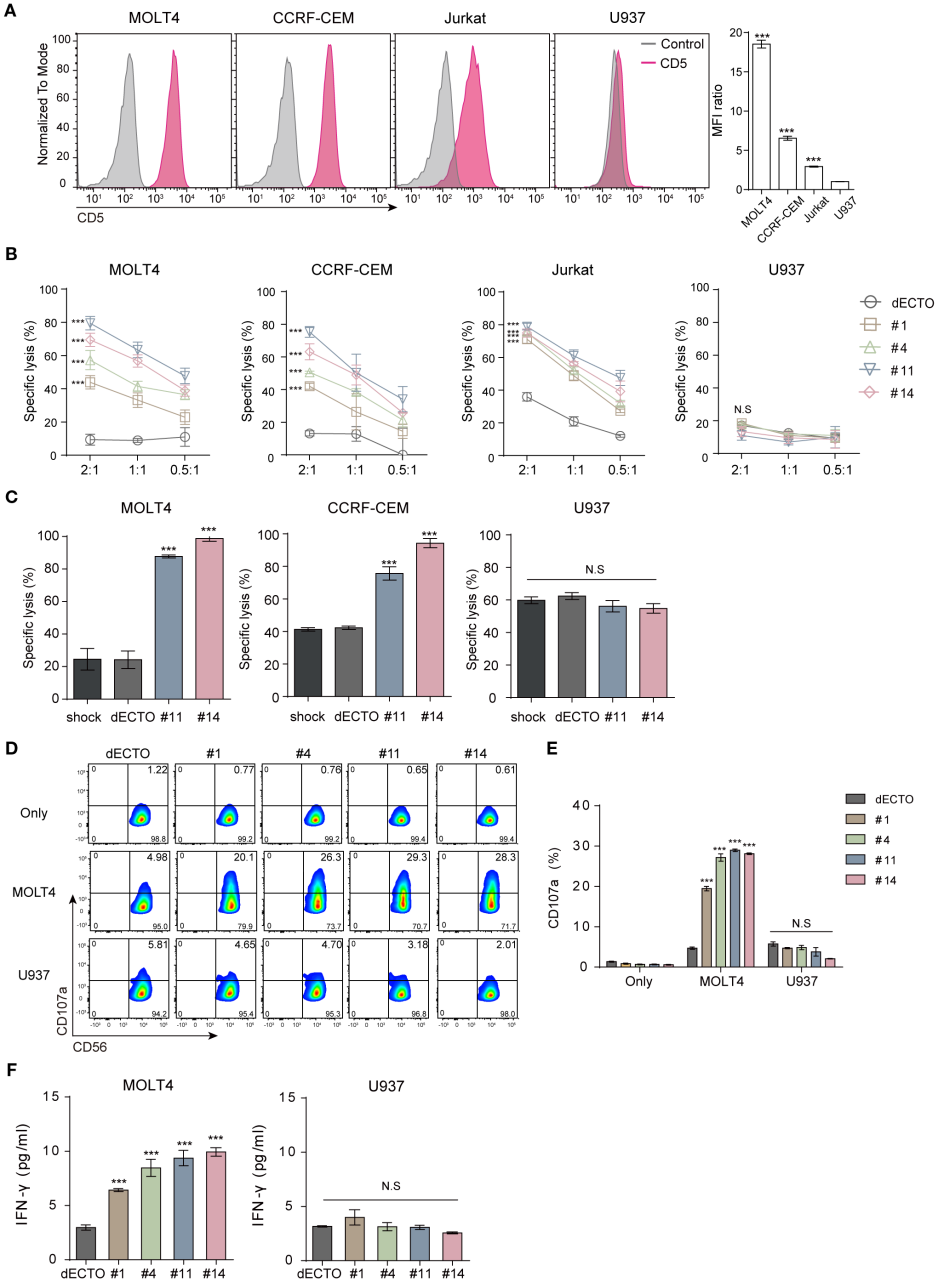
CD5 CAR-NK cells exhibit pronounced anti-cancer activity against CD5+ malignant T cells *in vitro*. **(A)** Flow cytometry analysis of CD5 expression on malignant T cell lines (MOLT4, CCRF-CEM, Jurkat) and lymphoma cell line (U937). Statistical significance was determined by one-way ANOVA with comparisons made relative to U937. **(B)** Cytotoxicity of four clones of CD5 CAR-NK cells co-incubated with CD5+ and CD5- cells for 2 hours at an E:T ratio of 2:1, 1:1, and 0.5:1. Statistical significance was determined using two-way ANOVA, and p values indicate differences relative to the dECTO control at an E:T ratio of 2:1. **(C)** Cytotoxicity of UCB-derived CAR-NK in leukemia cell line and MNC at an E:T ratio of 1:1. Results are from three independent experiments. Statistical significance was determined by one-way ANOVA. **(D, E)** Flow cytometry analysis of CD107a expression in CD56-marked CD5 CAR-NK cells following co-culture with CD5+/- cells and **(F)** Quantification of IFNγ secretion by ELISA. Data are from three independent experiments. Statistical significance was determined by one-way ANOVA. N.S, not significant; ***p < 0.001.

Hematological malignant cell lines were used as targets to assess the anti-cancer efficacy of CD5 CAR-NK cells. MOLT4, CCRF-CEM, and Jurkat cells that express CD5 at levels exceeding 90% were used as CD5+ targets. U937 cells were used as a negative control because they rarely express CD5 (**Figure 3A**). CD5 CAR-NK cells showed a significantly higher killing efficacy against CD5+ malignant cell lines than dECTO (**Figure 3B**). In particular, among the four types of CD5 CAR-NK cells, #11 and #14 showed relatively high anti-cancer activities.

To further validate these findings in a clinically relevant setting, we employed primary NK (pNK) cells differentiated from human UCB mononuclear cells (**Supplementary Figure 2A**). By *in vitro* transcription (IVT), we synthesized the mRNA of two different CD5+ CAR (#11 and #14) and transferred them to pNK cells for transient expression (**Supplementary Figure 2B**). The cytotoxicity of UCB-derived CAR-NK cells was consistent with our previous findings. These cells exhibited a high killing capacity against CD5+ malignant T cell lines while remaining unresponsive to CD5- cells (**Figure 3C**). This was further supported by increased CD107a expression and IFN-γ secretion upon co-culture with CD5+ target cells, confirming antigen-specific responses (**Figures 3D–F**). Lack of anti-cancer activity against CD5- U937 cells corroborates the antigen-specific response, serving as a negative control for CD5 targeting. Furthermore, to strengthen this observation, we conducted additional cytotoxicity assays using CD5-negative leukemia cell lines (THP-1 and MV4-11; **Supplementary Figure 1F, G**), which showed no significant differences in cytotoxicity compared to the control. Under the same conditions, CAR transduction efficiency varied among clones, with #11 and #14 showing higher activity, while #1 and #4 exhibited lower cytotoxicity and were excluded from further experiments.

### 3.3 Normal T cells are less susceptible to CD5 CAR-NK cells under adequate scFv and CAR expression

**Figure 4:**
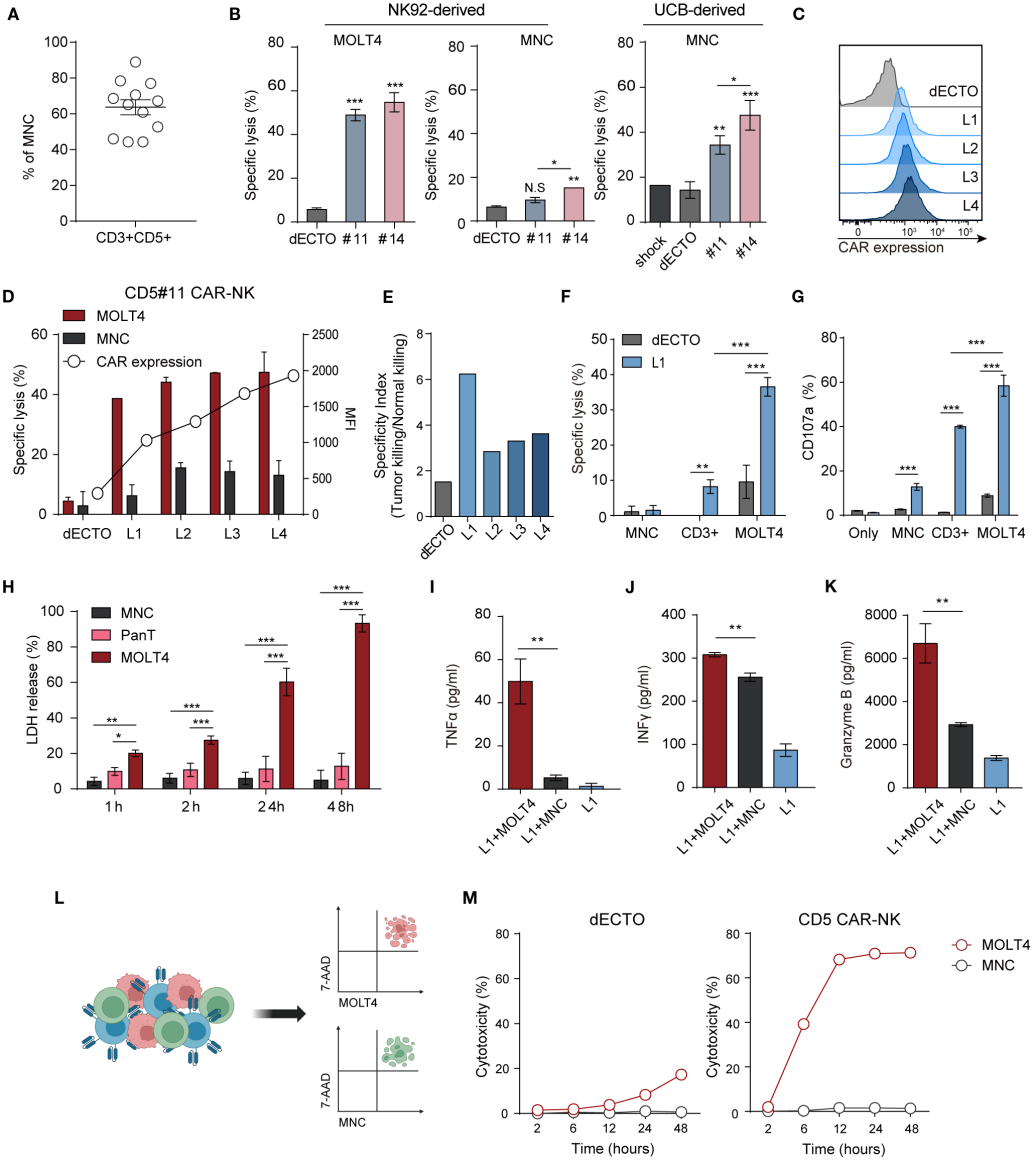
CD5 CAR-NK cells are less responsive to CD5+ normal T cells than malignant T cells. **(A)** The proportion of T cells expressing CD3 and CD5 in MNC derived from UCB accounts for 40–90%. Dots represent individual donors. **(B)** Cytotoxicity of two CD5 CAR-NK cells (#11 and #14), generated from NK92-derived and UCB-derived NK cells, against MOLT4 and MNC at an E:T ratio of 1:1. Statistical significance was determined by one-way ANOVA. **(C)** Flow cytometry analysis of monoclonal CAR-NK cells (L1-L5) with different CAR levels, derived from the less toxic CD5 CAR-NK cell line (#11). **(D)** Assessment of killing efficacy against MOLT4 and MNC by monoclonal CD5 CAR-NK cells with varying CAR expressions at an E:T ratio of 1:1. **(E)** Selectivity index (tumor killing/normal killing) of L1–L4 clones showing tumor vs. normal killing. **(F)** Comparative analysis of CD5 CAR-NK cell (L1) cytotoxicity against MOLT4, MNC, and CD3+ cells isolated from MNC using microbeads. **(G)** Degranulation (CD107a) of L1 upon interaction with each target cell was measured by flow cytometry. Statistical significance was determined by paired two-tailed Student’s t-test. **(H)** The LDH release profile, an indicator of cytotoxicity, during the co-culture of L1 with the target MOLT4 or non-targets of MNC and Pan T cells. Statistical significance was determined by two-way ANOVA. Secretion of inflammatory cytokines **(I)** TNFα, **(J)** IFNγ, and **(K)** cytotoxic granule granzyme B after co-culture of L1 with target MOLT4 or non-target MNC. Results are from three independent experiments repeated. Statistical significance was determined by paired two-tailed Student’s t-test. **(L)** Schematic representation of triple co-culture involving CD5 CAR-NK cells (L1), MOLT4, and MNC, alongside the gating strategy for flow cytometry analysis. Cells are labeled as follows: MOLT4 (cell tracker- deep red), MNC (cell trace-violet), CAR-NK (anti-hCD56-FITC). **(M)** Dead target cells (7AAD+) were calculated as % at each time point after co-culture with CD5 CAR-NK cells (L1). N.S, not significant; *p < 0.05; **p < 0.01; ***p < 0.001.

CD5 is expressed on normal T cells and the surface of hematologically malignant cells and can cause on-target and off-tumor effects (9). We analyzed the cytotoxicity against human MNC isolated from human UCB, comprising 40-90% CD5+ T cells (**Figure 4A**). To evaluate off-target effects, CAR-NK cytotoxicity was assessed against normal immune cells. While both #11 and #14 effectively eliminated malignant T cells, they exhibited reduced lysis of MNCs, with #11 showing the most favorable safety profile. Consistent with this result, UCB-derived CAR-NK cells transiently expressing CAR showed higher toxicity towards normal cells in #14 than in #11 (**Figure 4B**). Upon reacting each CAR with cancer and normal cells, the downstream signaling strength of #14 was observed to be greater than that of #11 (**Supplementary Figure 3**). A summary comparison of the key properties of clones #11 and #14 is provided in **Supplementary Table 1**, underscoring clone #11’s balanced profile for further optimization. Based on these findings, we identified #11 as the optimal scFv to minimize on-target off-tumor toxicity for OptiCAR-NK development and focused on further optimizing the expression of #11 CD5 scFv in subsequent experiments.

CAR expression affects the toxicity of antigen-positive cells. Monoclonal CAR-NK cells with different CAR levels (L1-L4) were established by single-cell sorting using #11 (**Figure 4C**) to regulate the killing ability of CD5 CAR-NK cells. Functional assays against malignant T cells and MNC revealed an increased killing efficacy and elevated CAR expression. Notably, even cells with the lowest CAR expression (L1 group) showed high anti-cancer activity, at approximately 38%, and low toxicity towards MNC, at approximately 6% (**Figure 4D**). To further assess the balance between efficacy and safety, we calculated a selectivity index (tumor killing/normal killing) for L1–L4, which confirmed that clones with lower CAR expression (L1) maintained a more favorable efficacy-safety balance (**Figure 4E**). Cytotoxicity and degranulation assays were conducted using L1 cells (**Figures 4F, G**) to compare the toxicity of CD5 CAR-NK cells towards cancer and normal cells. In addition to MNCs, which include diverse immune cell populations, we also used CD3+ cells isolated from the UCB or hPBMC CD3+ Pan T cells as a specific off-tumor control. Although cytotoxicity against pan-T cells was higher than MNCs, CD5 CAR-NK cells maintained strong selectivity by preferentially killing malignant T cells over normal T cells. The release of granules (CD107a) showed a similar trend as cytotoxicity. Toxicity upon prolonged exposure to CD5 CAR-NK cells was confirmed by the LDH assay (**Figure 4H**). CD5 CAR-NK cells exhibited a cytotoxic effect of over 60% against MOLT4 after 24 h, surpassing 90% within 48 h, while maintaining low toxicity (~10%) against MNCs and CD3+ Pan T cells. CD5 CAR-NK cells demonstrated anti-cancer efficacy against MOLT4 cells with no significant aggressiveness against normal human immune cells. Cytokine release assays confirmed selective activation, with IFN-γ, TNF-α, and granzyme B levels significantly elevated in malignant T cell co-cultures compared to normal cells (**Figures 4I–K**). Additionally, conditioned medium from L1-MOLT4 co-culture had no discernible effect on the viability of Raw264.7 macrophages, suggesting minimal off-target toxicity (**Supplementary Figure 4A**). These findings reinforce the therapeutic potential of OptiCAR-NK cells to eliminate malignant T cells while sparing normal immune cells.

Furthermore, to examine whether CD5 CAR-NK cells preferentially target cancer cells over normal cells when exposed simultaneously, we co-incubated with MOLT4, MNC, and CAR-NK cells at a ratio 1:1:1. Flow cytometry analysis showed a higher proportion of dead cancer cells compared to normal cells (**Figure 4L**). Notably, at later time points (up to 48 h), cytotoxicity against MOLT4 became sustained and more pronounced, whereas MNCs remained largely unaffected (**Figure 4M**). Similarly, in co-cultures with CD3+ T cells, CD5 CAR-NK cells exhibited selective cytotoxicity against malignant cells (**Supplementary Figure 4B**). These findings emphasize the reduced toxicity of CD5 CAR-NK cells towards normal cells, supporting #11 scFv and L1 expression density as the optimal conditions for achieving a balance between efficacy and safety in OptiCAR-NK.

### 3.4 Increased sensitivity of CD5 CAR-NK cells to malignant T cells is modulated by scFv, CAR expression, and differential ligand presentation

**Figure 5:**
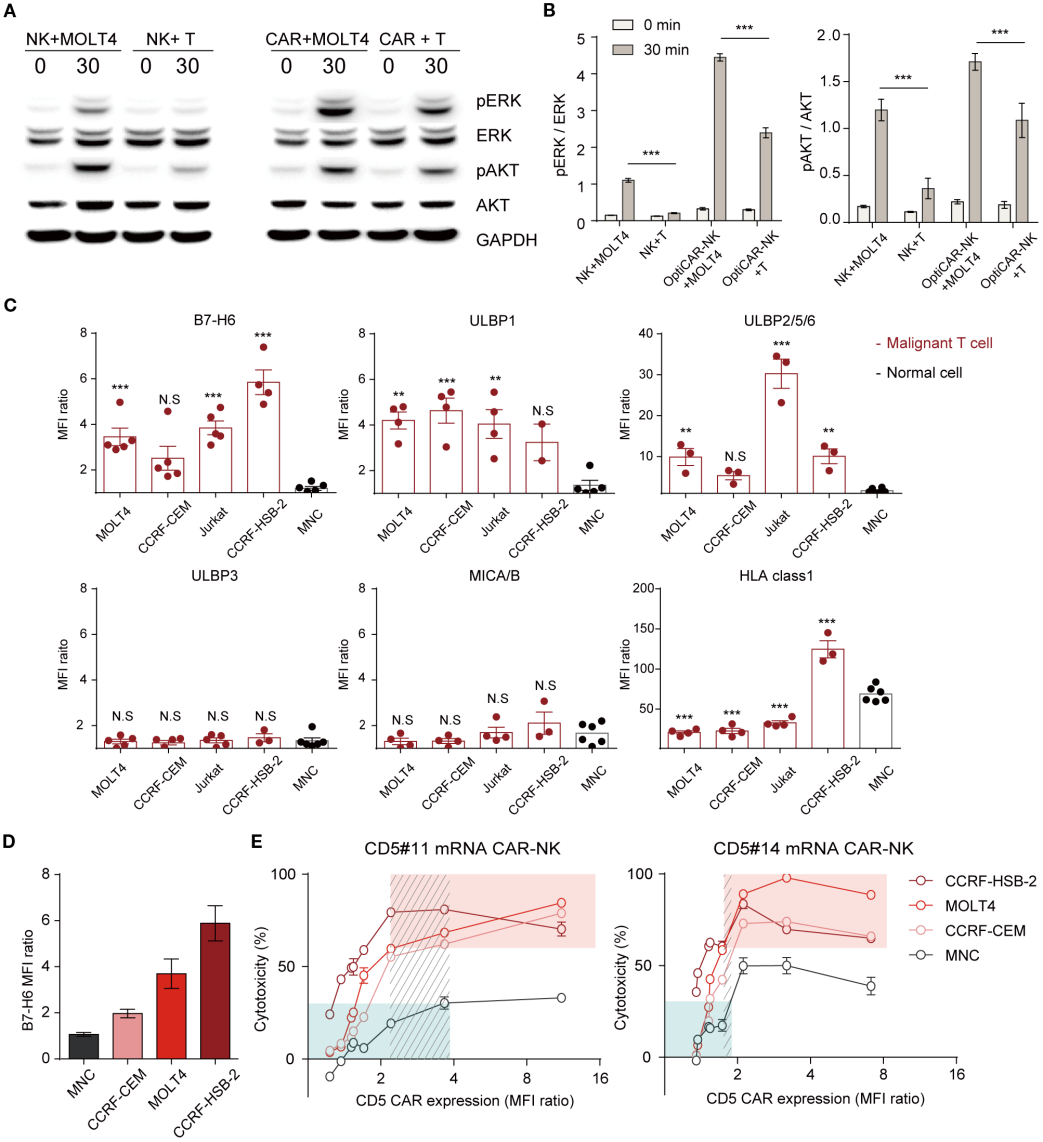
Differential CD5 CAR-NK activation in cancer and normal cells following scFv, CAR density, and ligand modulation. **(A)** Western blotting analysis of lysate from NK92 cells and CD5 CAR-NK cells co-incubated with MOLT4 or MNC at an E:T ratio of 10:1. **(B)** Protein levels were quantified as the relative band density. Data are from three independent experiments. Statistical significance was determined by two-way ANOVA. **(C)** Flow cytometry analysis for activating ligands (B7-H6, ULBP1, ULBP2/5/6, and MICA/B) and expression of inhibitory ligands on malignant T cell lines (MOLT4, CCRF-CEM, Jurkat, and CCRF-HSB-2) and MNC. Results are representative of three independent experiments replicated at least three times. Statistical significance was determined by one-way ANOVA with comparisons made relative to MNC. **(D)** Graph of malignant T cell lines and MNC ranked by B7-H6 expression. **(E)** Comparative cytotoxicity based on CAR level modulation using mRNA of CD5 CAR-NK (#11 and #14) at an E:T ratio of 1:1. Data represent mean ± standard error of the mean (SEM). N.S, not significant; *p < 0.05; **p < 0.01; ***p < 0.001.

Despite expressing the same antigen, normal cells exhibited reduced responsiveness to CAR-NK cells, prompting an investigation into the underlying mechanisms. NK92 cells inherently showed slightly increased phosphorylation of the key activation signaling proteins, AKT and ERK, in response to cancer cells compared with normal cells. However, the signal strength was insufficient to effectively eradicate cancer cells (**Figures 3**, **4**). CAR expression enhanced activation signals in both normal and malignant targets, with a significantly stronger response in cancer cells (**Figures 5A, B**). However, signal transduction remained notably higher in cancer cells than in normal cells.

The activation and effector functions of NK cells are contingent on the integration of signals derived from two distinct classes of receptors, activating and inhibitory (40, 41). To understand this selective activation, we analyzed activating and inhibitory ligand expression in target cells B7-H6, the primary ligand for NKp30, was significantly upregulated in cancer cells but nearly absent in normal cells (**Figure 5C**; **Supplementary Figure 5A**). The ligands that bind to the NK-activating receptor NKG2D are ULBP1 and ULBP2/5/6, expressed at higher levels in cancer cells than in normal cells. However, NKG2D ligands, ULBP3 and MICA/B, showed similar expression levels in cancer and normal cells. Downregulation of B7-H6 in MOLT4 and Jurkat cells led to reduced CAR-NK reactivity, confirming its role in modulating cytotoxicity (**Supplementary Figures 5B, C**). During co-culture with the target cells, we monitored the alterations in B7-H6 and CD5 expression levels in the surviving target cells by flow cytometry (**Supplementary Figures 5D, E**). The results demonstrated a progressive reduction in the B7-H6 levels in the remaining target cells (MOLT4). These findings indicate that CAR-NK cells preferentially target cells with elevated B7-H6 expression. Likewise, CD5 levels gradually declined, with a more rapid reduction in cancer cells exhibiting high B7-H6 expression. These results clearly demonstrate that the cytotoxic activity of CD5 CAR-NK is influenced by B7-H6 expression on target cells. The responsiveness of CAR-NK cells to antigen-expressing targets is primarily influenced by CAR expression levels and target cell ligand presentation. To assess the impact of CAR intensity, we fine-tuned CAR expression using mRNA transfection and evaluated its efficacy against targets with varying B7-H6 levels (**Figure 5D**). Cytotoxicity correlated with CAR expression, increasing proportionally before plateauing and eventually declining beyond a threshold (**Figure 5E**). Furthermore, cells with higher B7-H6 expression exhibited greater susceptibility at equivalent CAR levels. At the same CAR expression level, CD5 #14 showed stronger lysis activity than CD5 #11, consistent with prior findings (**Figure 3B**; **Supplementary Figure 2**). To indicate the balance between efficacy and safety, we highlighted a selective range where malignant cell killing was relatively high (>60%; red box) and normal cytotoxicity remained low (<30%; green box). Within this range, #11 CAR-NK maintained broader selectivity compared to #14, which exhibited a narrower range. To further quantify this relationship, we calculated a selectivity index (tumor killing/normal killing) using the same dataset. As shown in **Supplementary Figure 6**, the selectivity index increased with CAR expression levels up to an optimal range but declined when expression became excessive, underscoring the importance of both scFv selection and CAR expression tuning in defining optimized CAR-NK cells. These results demonstrate that CAR-NK activity is modulated by differential ligand expression and CAR expression levels, emphasizing the need for precise tuning to maximize efficacy while minimizing off-target effects.

### 3.5 OptiCAR-NK cells achieve significant tumor suppression and prolonged survival in the T cell leukemia xenograft model

**Figure 6:**
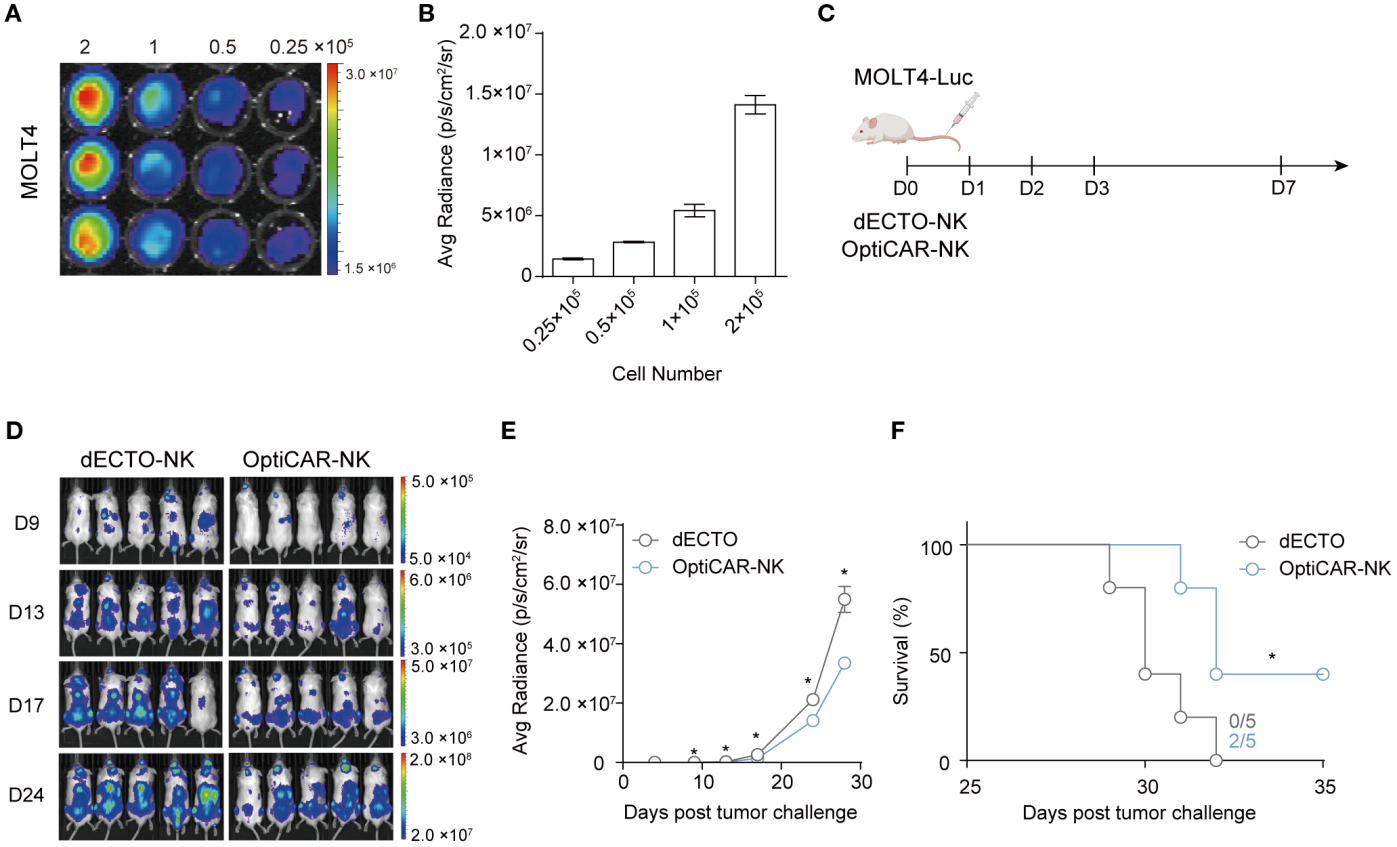
OptiCAR-NK regulates the systemic progression of T-ALL in a mouse xenograft model. **(A)** Bioluminescence imaging (BLI) of MOLT4-Luc. MOLT4-Luc cells were seeded from 2×10^5^/100 μl down to 0.25×10^5^/100 μl in a two-fold serial dilution and 150 μg/mL D-Luciferin was added to each well and incubated for 10 minutes. **(B)** Graphical correlation between bioluminescence signals and cell numbers. **(C)** Schematic diagram of *in vivo* experiment. NOG mice (n=5 per group) were injected intravenously with 3×10^5^ MOLT-Luc followed by intravenous injection of 3×10^6^ dECTO or OptiCAR-NK for 5 times. **(D)** Tumor burden was monitored throughout the 4 weeks by quantifying luminescence signals through IVIS imaging. **(E)** Statistical analysis of the bioluminescence intensity of each group measured at 24 days post-tumor inoculation. Each dot represents an individual animal. Statistical significance was determined by paired two-tailed Student’s t-test. Data represent mean ± SEM. **(F)** Kaplan-Meier survival curve of mice (n=5 per group) injected with dECTO or OptiCAR-NK cells. Statistical significance was determined using a log-rank (Mantel–Cox) test. *p < 0.05.

Having established the anti-cancer efficacy of OptiCAR-NK against T cell hematological malignancies *in vitro*, we validated their anti-cancer properties *in vivo* using a NOG xenograft model. We established a MOLT4 cell line expressing firefly luciferase (MOLT4-Luc), exhibiting a robust positive correlation between firefly luciferase activity and cell number (**Figure 6A**). MOLT4-Luc was injected intravenously on D0 to investigate the effect of OptiCAR-NK cells on controlling the progression of T cell leukemia *in vivo*. Subsequently, dECTO and OptiCAR-NK were intravenously injected five times (**Figure 6B**). The tumor burden was monitored using bioluminescence imaging every 3–4 days. Consequently, the OptiCAR-NK-infused group exhibited notable inhibition of tumor progression and an extended survival period compared with the control group (**Figures 6C–F**).

### 3.6 OptiCAR-NK cells exhibit negligible on-target off-tumor toxicity in humanized mouse model

**Figure 7:**
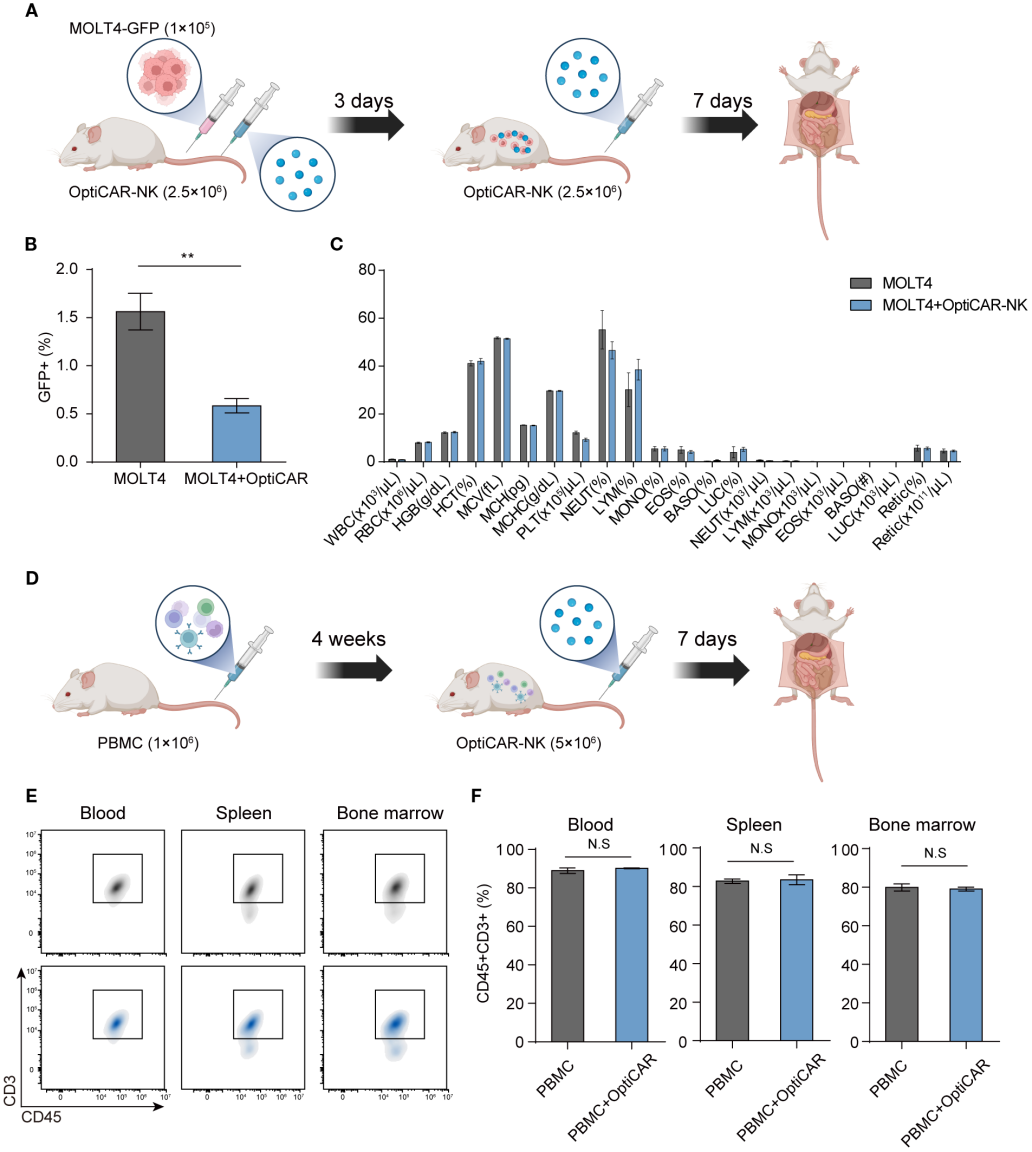
OptiCAR-NK cells effectively reduce tumors with no adverse impact on blood components and normal T cells. **(A)** Schematic of experiments for investigating the effect of OptiCAR-NK (2.5×10^6^, 2 times) treatment on the elimination of GFP-tagged MOLT4 (1×10^5^) within the xenograft model (n=4 per group) and for analyzing changes in blood cell compositions. **(B)** Flow cytometry analysis for the percentage of GFP+ cells in the bloodstream following sole injection of MOLT4 and combined injection of MOLT4+ OptiCAR-NK at day 10 post-inoculation of cancer, and **(C)** blood composition analysis. **(D)** Schematic depicting the experimental protocol to assess the preservation of human T cells following L1 (2.5×10^6^) injection in PBMC (1×10^6^)-engrafted humanized mice (n=3 per group). **(E, F)** The ratio of human CD45+CD3+ T cells in the blood, spleen, and bone marrow was analyzed by flow cytometry after administering OptiCAR-NK injection in PBMC-engrafted humanized mice. Data represent mean ± SEM. Statistical significance was determined by paired two-tailed Student’s t-test. N.S, not significant; **p < 0.01.

We conducted an experiment involving the administration of GFP-tagged MOLT4 and OptiCAR-NK cells to NOG mice (**Figure 7A**) to evaluate the *in vivo* anti-cancer effect of OptiCAR-NK cells on MOLT4 and the potential risks of the therapy. The percentage of GFP+ cells within the collected blood was higher in the group where only MOLT4 was injected (MOLT4 group) than in the MOLT4+L1 group, demonstrating the anti-cancer effect of the therapy (**Figure 7B**). The MOLT4 and MOLT4+L1 groups did not exhibit statistically significant differences in various blood components, including white blood cells (WBC), red blood cells (RBC), and hemoglobin (HGB) (**Figure 7C**). This outcome suggests the safety of OptiCAR-NK, either by itself or by its anti-cancer activity against MOLT4, with no significant impact on blood components. OptiCAR-NK was applied to humanized immune mice with engrafted hPBMCs for further validation of safety (**Figure 7D**). This experiment was conducted to examine the effects of OptiCAR-NK cells on normal human T cells *in vivo*. Analysis of human CD45+CD3+ cell ratios in samples obtained from the blood, spleen, and bone marrow revealed no statistically significant differences between the group of mice with engrafted PBMCs only (PBMC group) and the group injected with OptiCAR-NK cells (PBMC+OptiCAR) (**Figures 7E, F**). This confirmed that OptiCAR-NK cells did not exert adverse effects, including removal capability, on normal human CD45+ T cells among the engrafted PBMCs in NOG mice. We observed the effect of OptiCAR-NK dosage on the general symptoms of NOG mice (**Supplementary Figure 6A**). There were no statistically significant differences in the body weight between the untreated mice (control group) and the group intravenously injected with OptiCAR-NK (OptiCAR-NK group) in both male and female animals. No differences attributable to OptiCAR-NK treatment were found in the weights of the major organs associated with toxicity, such as the liver and lungs, upon necropsy (**Supplementary Figure 6B**). These observations, along with the absence of significant changes in blood components as confirmed through blood tests, indicate that this therapy has a low potential to induce toxicity (**Supplementary Figure 6C**).

## 4 Discussion

CAR-T cells have shown remarkable effectiveness against CD19-expressing B cell hematologic cancers, as demonstrated by the Food and Drug Administration (FDA) approval of Novartis’ Kymriah and Gilead’s Yescarta in 2017 (42, 43). However, employing autologous T cells in CAR-T therapies for T cell hematologic malignancies poses various challenges because of the phenotypic resemblance between cancer and normal cells. As an alternative solution to address this challenge, using NK cells with a distinct antigenic profile from T cells has been suggested.

CD5 is recognized as a marker of malignant T cells; however, it is also expressed in various cell types, including T cells and B cells (44, 45). Therefore, the ability of CD5-targeting CAR-NK cells to identify malignant T cells and other CD5-expressing cells is crucial. Previous research on CD5 CAR-NK cells has proposed various strategies to mitigate side effects, including regulating NK cell dosage and frequency, employing short-term activated NK cells to prevent long-term aplasia, and combining with ASCT after achieving complete remission (46). More recently, safety switch systems such as HSV-TK have been explored (47). While these systems offer an emergency safeguard, they function only as a *post hoc* control and do not fundamentally alter CAR behavior toward normal T cells. In contrast, our study aimed to develop OptiCAR-NK cells that integrate efficacy and safety at the design stage by fine-tuning scFv affinity and CAR expression levels, thereby reducing off-tumor toxicity while preserving anti-tumor activity. Indeed, this strategy enabled selective elimination of malignant T cells while sparing normal CD5+ cells.

Given the importance of selective targeting in CAR-NK therapy, we further investigated factors influencing NK cell activation, focusing on B7-H6, a key ligand involved in NKp30-mediated cytotoxicity. B7-H6 is specifically expressed in cancer cells and serves as a biomarker and therapeutic target for malignant T cells (48, 49). In a comparative analysis of human NK cell activation induced by NKp30 or NKG2D, Andre et al. evaluated anti-NKG2D and anti-NKp30. Their findings revealed that upon treatment with anti-NKp30, NK cells exhibited higher activation, proliferation, and cytotoxicity compared to anti-NKG2D (50). Recent studies further indicate that interactions between NK cells and the B7-H6 ligand exert a much stronger effect in maximizing NK cell activation compared to other ligands such as MICA/B or ULBPs (37). Building on previous research, we focused on the regulation of B7-H6 to observe changes in CAR-NK cell activity. Fine-tuning CAR expression using mRNA modulation revealed the following tendency in malignant T cell lines: higher B7-H6 levels corresponded to lower CAR levels, where cytotoxicity reached saturation. However, even in normal cells lacking B7-H6, cytotoxicity gradually increased with increasing CAR levels (**Figures 3D–F**). This approach underscores the importance of optimizing CAR expression levels to harness NK cells’ innate ability to distinguish cancer cells from normal cells while minimizing the risk of on-target off-tumor cytotoxicity. Although B7-H6 was used as a representative ligand in this study, modulating other ligands is also expected to produce similar effects with B7-H6. Previous studies have shown that the modulation of other ligands influences NK cell activity, suggesting that such regulation could similarly affect the activity of CAR-NK cells (37). In addition to ligand interactions, CAR activity is significantly affected by intrinsic factors such as scFv properties and CAR expression levels, which play a crucial role in optimizing therapeutic efficacy and safety.

In **Figure 5E**, both CAR expression levels and scFv type significantly influenced the lysis activity of CAR-NK cells. Notably, #14 CD5 CAR-NK exhibited a sharp increase in toxicity against antigen-expressing targets even at low CAR expression levels, suggesting potential challenges in fine-tuning CAR expression with this scFv. As shown in **Supplementary Figure 3**, scFv type also affected downstream signaling strength, with #11 and #14 CAR-NK cells exhibiting distinct activation signals against the same target. Additionally, CD5#14 CAR demonstrated significantly higher binding to CD5+ target cells, contributing to its superior lysis activity but also increasing the risk of on-target off-tumor toxicity. In contrast, CD5#11 showed a more balanced profile, achieving strong anti-cancer efficacy with lower toxicity. These findings highlight the importance of binding efficiency in determining both efficacy and safety in CAR therapies and align with previous studies suggesting that low-affinity scFv can help mitigate off-target effects (21–23, 51, 52). Further characterization of scFvs is needed to fully understand these differences. Future studies should include kinetics and affinity assays to determine binding properties, as well as domain mapping to analyze structural variations in complementarity-determining regions (CDRs). These insights will help refine CAR-NK therapies to maximize efficacy while minimizing adverse effects. Consistent with this, our selectivity index analysis (**Supplementary Figure 6**) demonstrated that the efficacy–safety balance is jointly shaped by both scFv properties and CAR expression levels. For example, #11 CAR-NK maintained broader selectivity than #14, highlighting the need to optimize both parameters in defining an “optimized” CAR-NK.

Excessively high levels of CAR expression can trigger the self-aggregation of scFv, inducing tonic signaling, which contributes to exhaustion and functional decline of CAR-T cells, which exhibit markedly poor clinical responses (30, 31). Safety and effectiveness often present at opposite ends of the spectrum, and achieving an optimal balance between them is paramount for achieving the most favorable outcome. To ensure optimal CAR-NK function, we aimed to regulate CAR expression appropriately.

Our study demonstrated that OptiCAR-NK cells effectively targeted T-ALL cell lines while maintaining selective safety in UCB-MNCs and PBMCs. However, a limitation of this study is the lack of patient-derived primary T-ALL cells. Future research should incorporate these cells to further validate the efficacy and safety of CD5 CAR-NK therapy.

Optimizing CAR signaling is essential for improving the safety of T-cell hematological malignancy treatments. While these findings provide a foundation for advancing CAR-NK therapies, they should be interpreted with caution, as the optimal level of CAR expression can vary depending on various factors, such as cell type, target antigen profile, and individual patient conditions.

In conclusion, fine-tuning scFv and CAR expression is key to balancing CAR-NK activity, ensuring efficient tumor elimination while minimizing toxicity. Furthermore, selecting an appropriate scFv and regulating CAR expression is critical for harnessing the inherent ability of CAR-NK cells to distinguish cancer cells from normal cells. OptiCAR-NK represent a potential strategy for CAR therapies aimed at balancing anti-cancer efficacy with safety.

## Data Availability

The original contributions presented in the study are included in the article/**Supplementary Material**. Further inquiries can be directed to the corresponding author.
