# Supplementary material for: Fine-tuning signal strength in CD5 CAR-NK cells for targeted T cell cancer therapy
Source: Front Immunol. 2025 Sep 19;16:1674376. doi: 10.3389/fimmu.2025.1674376 (PMC12491253; doi:10.3389/fimmu.2025.1674376)
Supplement: Supplementary file 1 [file DataSheet1.pdf]

Supplementary file

Fine-Tuning Signal Strength in CD5 CAR-NK Cells for Targeted T Cell Cancer Therapy

Seona Jo, Yu Bin Lee, Seok-Min Kim, Soo Yun Lee, Myeongjin Choi, Mi-lang Kyun, Seo Yule Jeong, Sunyoung Lee, Ji Hyun Kim, Yoonji Kim, Yu Jung Kim, Sora Park, Kyoung-Sik Moon and Tae-Don Kim

Supplementary Figures 1-7

Supplementary Table 1

Supplementary Figure 1

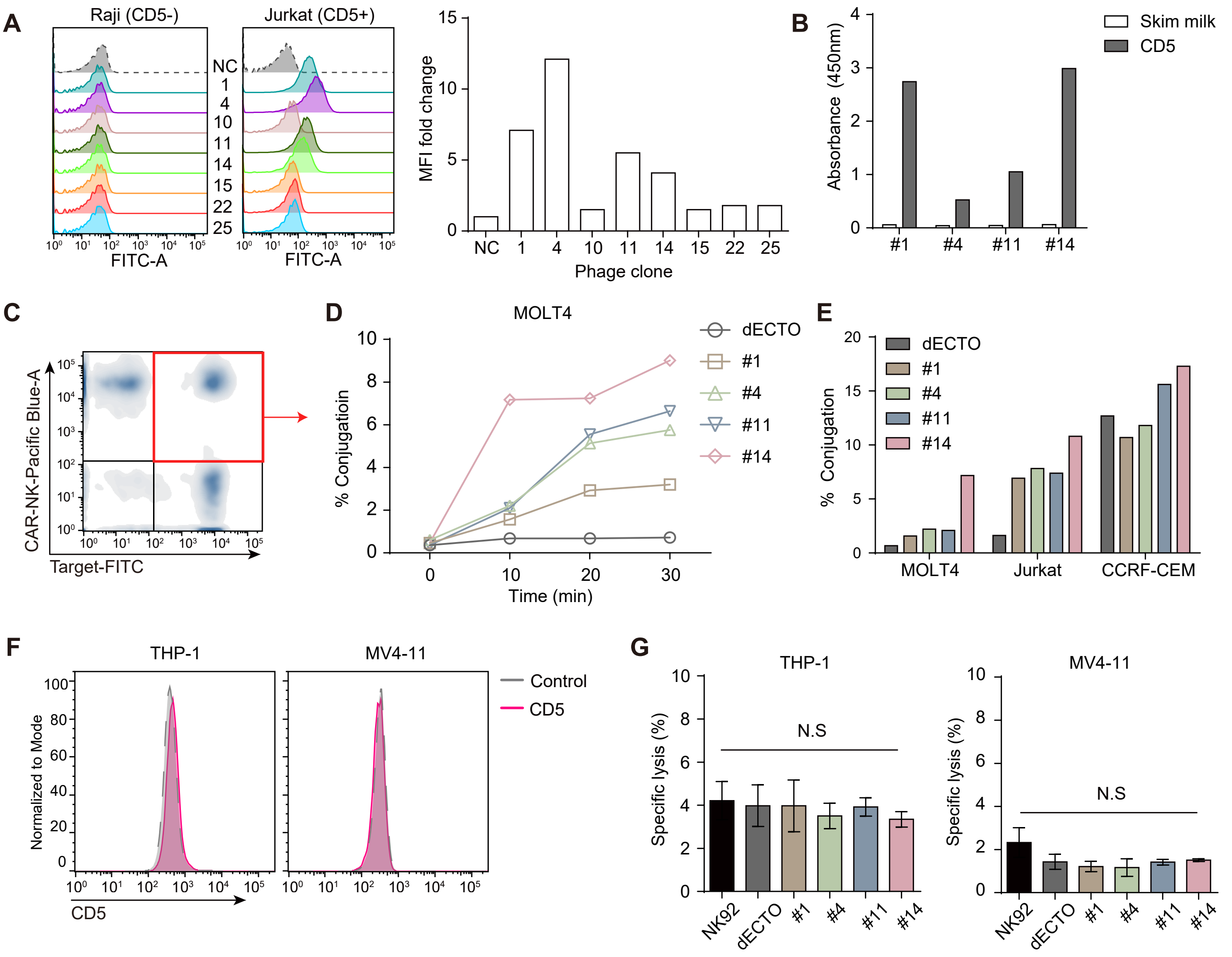

Figure S1. Screening anti-CD5 scFvs for binding to the T cell leukemia cell line

(A) Flow cytometry assessment of CD5 scFv express phage cell binding efficiency to CD5 in Raji and Jurkat cells, comparing mean fluorescence intensity (MFI) fold changes to NC. NC: helper pharge+anti-M13 antibody. (B) Four clones of the phage display library were tested in a monoclonal phage ELISA against CD5. For negative controls, uncoated wells were blocked with skim milk to provide the background absorbance signal. (C) Cells were labelled as follows: tumor cells(calcein), CD5 CAR-NK cells(cell trace-violet) and double positive population indicates conjugation. (D) Conjugation population of MOLT4 and CAR-NK cells at indicated time points after co-culture. (E) Conjugation population of target cells and CAR-NK cells at 20min after co-culture. (F) Flow cytometry analysis for detecting the expression of CD5 on leukemia cell line. (G) Cytotoxicity of CD5 CAR-NK cells in leukemia cell line at an E:T ratio of 2:1. Results are from three independent experiments. Statistical significance was determined by one-way ANOVA.

Supplementary Figure 2

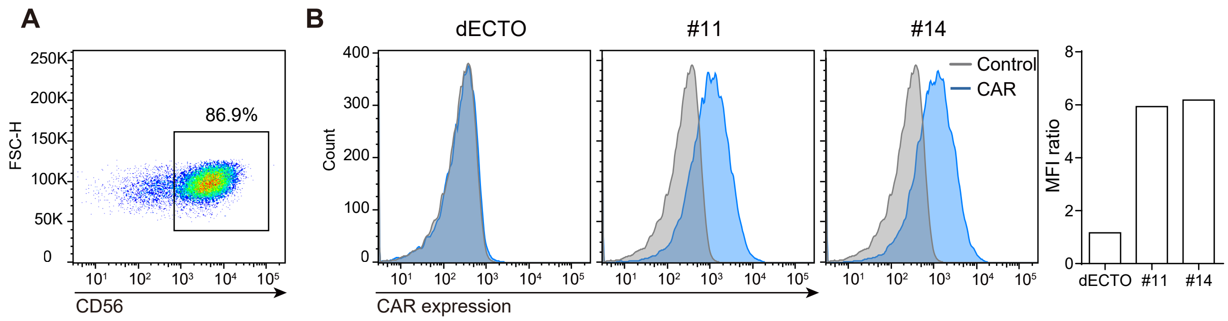

Figure S2. mRNA-based CD5 CAR-pNK shows superior antigen-specific anti-cancer effects in vitro.

(A) The frequency of CD56 expression on NK cells assessed by flow cytometry after 8 days of cytokine-induced differentiation from MNC. (B) Flow cytometry analysis for detecting the expression of CD5 CAR on NK cells following transfection with mRNA encoding CD5 CAR via electroporation.

Supplementary Figure 3

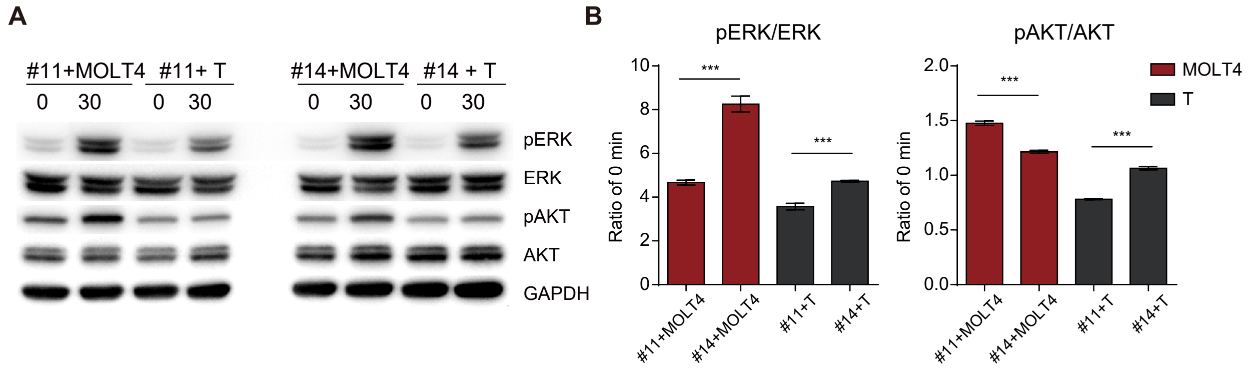

Figure S3. scFv specificity alters signal intensity in CAR-NK Cells

(A) Western blotting analysis of lysate from #11 and #14 CAR-NK co-incubated with MOLT4 or MNC at an E:T ratio of 10:1. (B) Protein levels were quantified as the relative band density. Statistical significance was determined by paired two-tailed Student's t-test.

Supplementary Figure 4

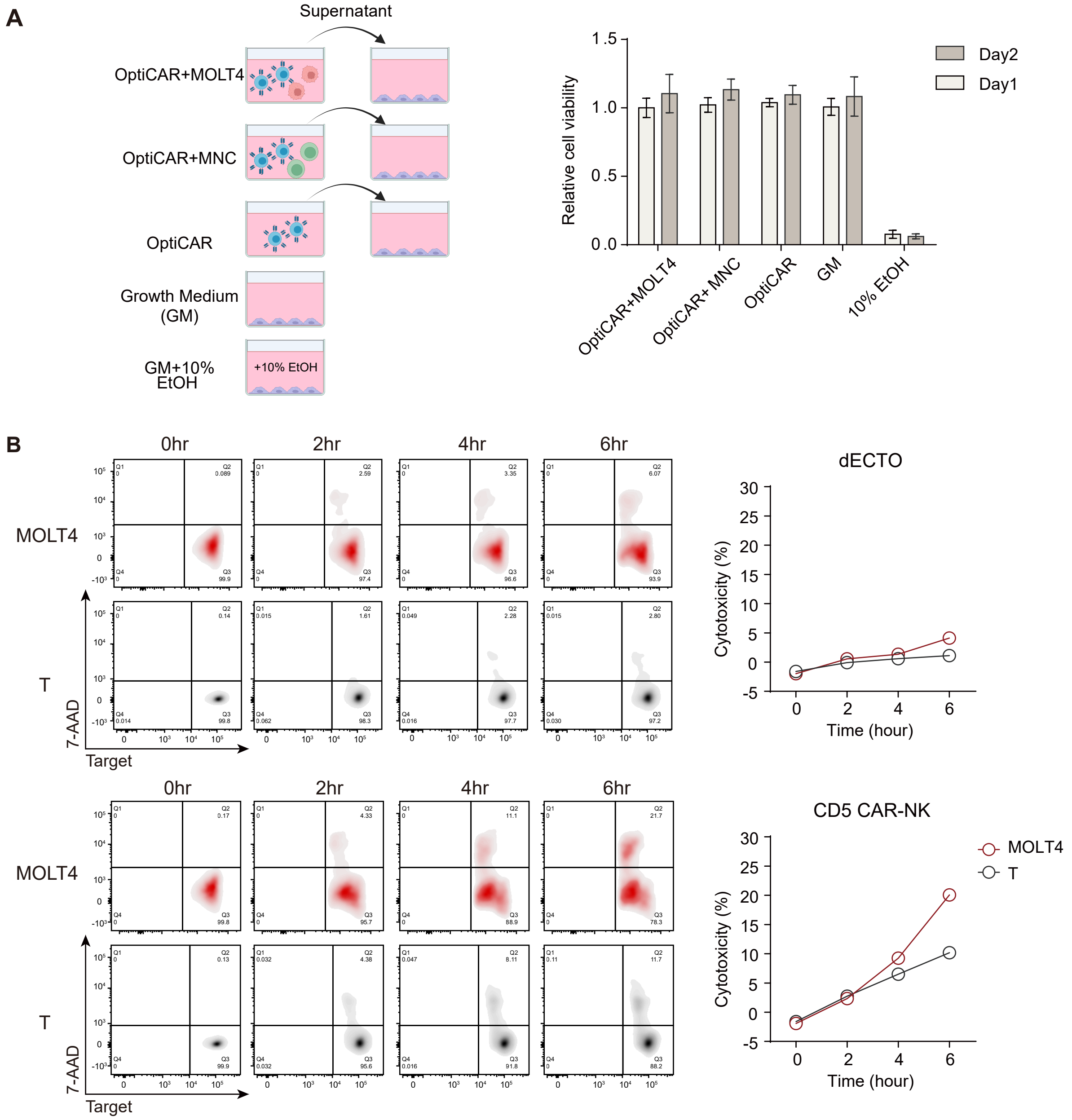

Figure S4. OptiCAR-NK cells exhibit reduced reactivity towards normal cells compared to cancer cells. (A) Relative cell viability of macrophage cells treated with conditioned mediums obtained after co-culture of OptiCAR-NK with target MOLT4 or non-target MNC. (B) dECTO and CAR-NK cells were labeled with anti-human CD56-FITC. Dead cells (7AAD+) in each target cell group were calculated as % at each time point after co-culture with OptiCAR-NK

Supplementary Figure 5

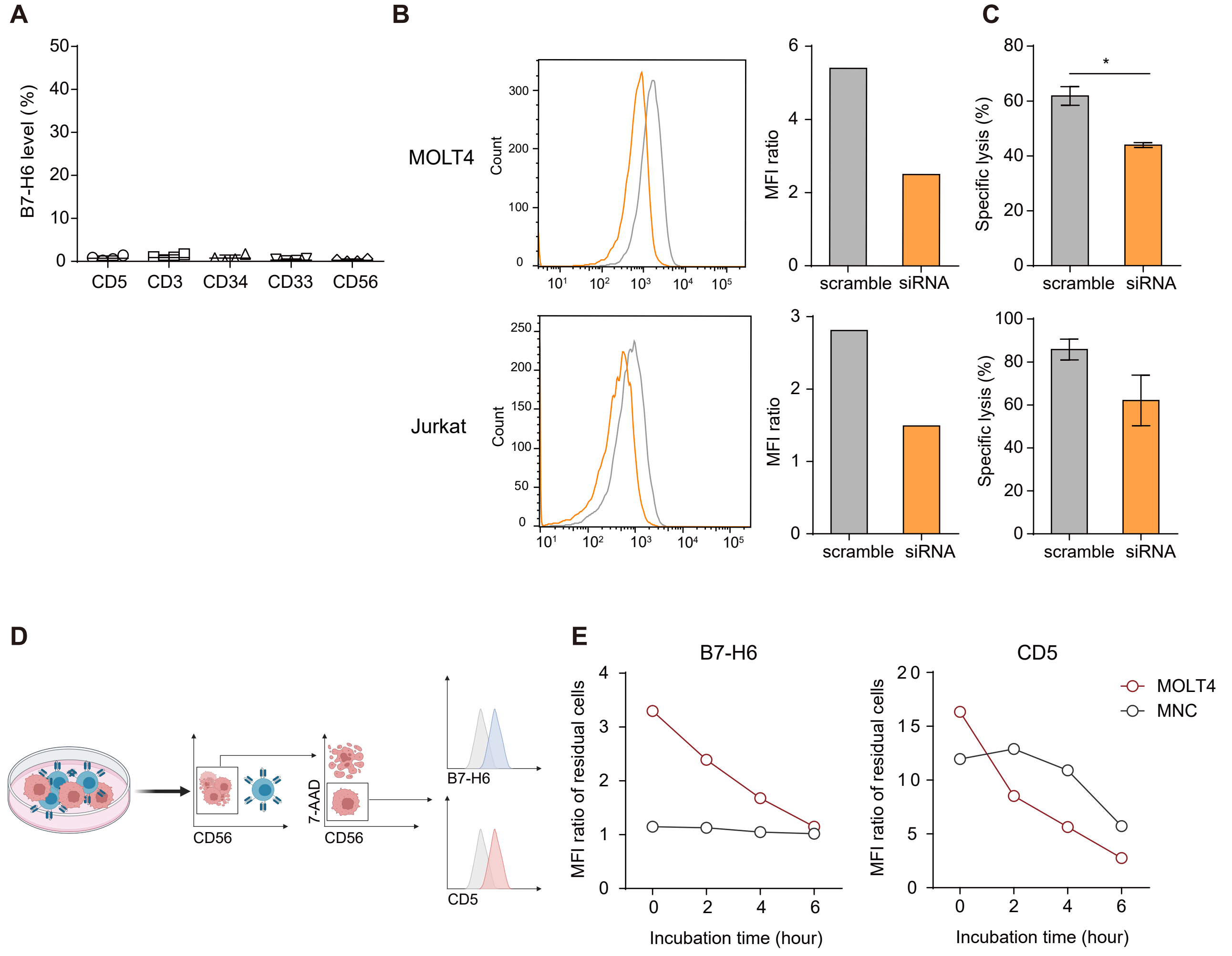

Figure S5. The activity of OptiCAR-NK cells varies based on the expression of B7-H6 in cancer cells.

(A) Expression levels of B7-H6 across various blood components derived from cord blood using cell surface markers. CD5: T cell or B cell, CD3: T cell, CD34: Hematopoietic stem cell, CD33: myeloid cell, CD56: NK cell. (B) Representative surface expression of B7-H6 on MOLT4 and Jurkat cells post-transfection with scrambled siRNA (scramble) or B7-H6 siRNA(siRNA). (C) Cytotoxicity was evaluated by co-culturing CD5 CAR-NK cells with transfected target cells in a 1:1 ratio. Data are from three independent experiments. Statistical significance was determined by paired two-tailed Student's t-test. (D) Schematic illustration of co-culture followed by flow cytometry analysis. OptiCAR-NK cells were co-cultured with each target cell (MOLT4 and MNC) and levels of B7-H6 and CD5 were detected in surviving target cells over time. (E) Graph depicting the mean fluorescence intensity (MFI) ratio of B7-H6 (right panel) and CD5 (left panel) in residual cells.

Supplementary Figure 6

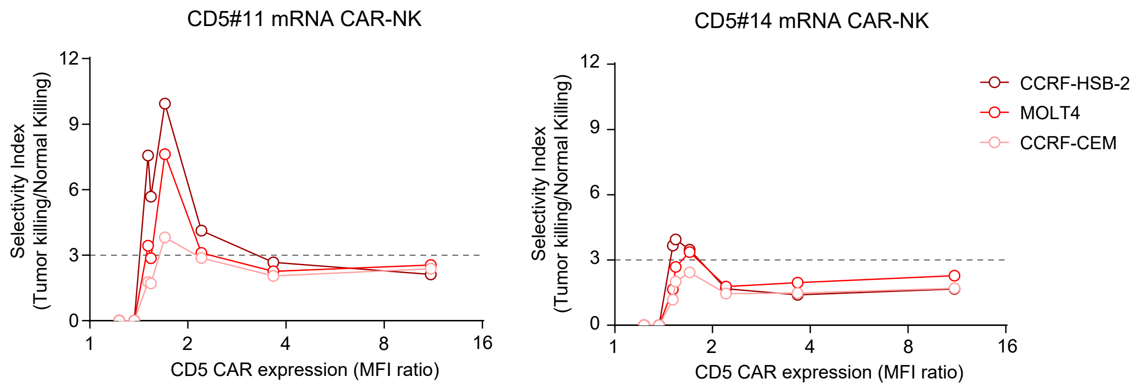

Figure S6. Selectivity index analysis of CD5 CAR-NK cells.

The ratio of tumor cell killing to normal cell killing (safety index) was analyzed in CD5#11 and CD5#14 mRNA CAR-NK cells. The safety index is shown in relation to CAR expression levels (MFI ratio) using CCRF-HSB-2, MOLT4, and CCRF-CEM as representative CD5+tumor targets.

Supplementary Figure 7

A

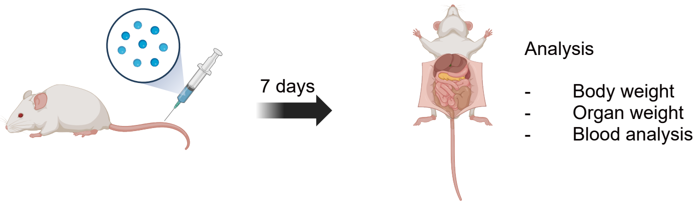

B

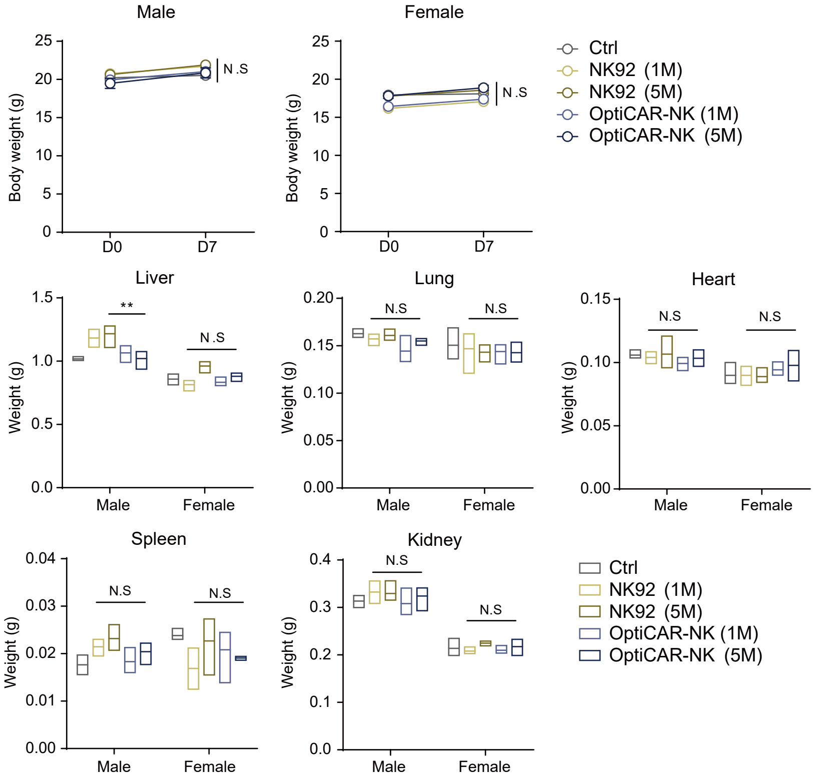

C

Blood components \ Groups	Male					Female				
	Ctrl	NK92 (1M)	NK92 (5M)	OptiCAR (1M)	OptiCAR (5M)	Ctrl	NK92 (1M)	NK92 (5M)	OptiCAR (1M)	OptiCAR (5M)
WBC (×10³/μL)	1.2 ± 0.4	1.0 ± 0.1	1.0 ± 0.1	1.1 ± 0.0	0.8 ± 0.1	0.7 ± 0.2	0.7 ± 0.2	1.1 ± 0.5	0.8 ± 0.2	0.7 ± 0.1
RBC (×10⁶/μL)	9.6 ± 0.2	9.8 ± 0.0	9.8 ± 0.3	9.6 ± 0.2	10.0 ± 0.3	9.3 ± 0.3	9.4 ± 0.1	9.1 ± 0.1	9.0 ± 0.5	9.2 ± 0.1
HGB (g/dL)	14.5 ± 0.4	14.5 ± 0.2	14.4 ± 0.3	14.3 ± 0.5	14.8 ± 0.5	14.1 ± 0.4	14.1 ± 0.3	13.6 ± 0.2	13.5 ± 0.3	13.8 ± 0.1
HST (%)	52.1 ± 1.4	52.0 ± 0.3	51.9 ± 1.2	51.6 ± 0.9	5.5 ± 1.8	49.5 ± 1.4	49.3 ± 0.6	47.7 ± 0.8	47.7 ± 2.2	45.7 ± 1.2
MCV (fL)	54.0 ± 0.6	53.0 ± 0.5	53.1 ± 0.4	53.6 ± 0.2	53. ± 0.3	53.2 ± 0.5	52.6 ± 0.1	52.3 ± 0.9	52.8 ± 0.3	53.3 ± 0.6
MCH (pg)	15.0 ± 0.3	14.8 ± 0.3	14.7 ± 0.1	14.8 ± 0.3	14.8 ± 0.1	15.1 ± 0.1	15.0 ± 0.1	14.7 ± 0.3	15.0 ± 0.2	15.0 ± 0.2
MCHC (g/dL)	27.8 ± 0.3	27.9 ± 0.3	27.7 ± 0.1	27.7 ± 0.6	27.6 ± 0.2	28.4 ± 0.1	28.6 ± 0.3	28.6 ± 0.1	28.4 ± 0.2	28.2 ± 0.6
PLT (10³/μL)	1276.7± 205.3	1489.7 ± 88.2	1352.7 ± 70.5	1268.7 ± 28.4	1380.7 ± 10.8	1172.3 ± 141.9	1004.0 ± 96.4	1088.3 ± 79.8	1148.0 ± 91.0	1234.3 ± 35.2
NEUT (%)	70.2 ± 2.9	67.5 ± 5.5	67.7 ± 3.4	67.6 ± 5.4	63.4 ± 4.2	62.2 ± 8.1	71.5 ± 3.4	68.1 ± 8.4	73.2 ± 4.	65.0 ± 7.1
LYM (%)	17.5 ± 2.0	20.4 ± 3.7	19.9 ± 2.2	18.3 ± 3.3	21.1 ± 1.1	23.8 ± 7.0	15.4 ± 2.3	19.7 ± 7.2	14.4 ± 3.4	20.8 ± 6.9
MONO (%)	6.0 ± 1.5	9.1 ± 1.9	7.8 ± 2.0	8.3 ± 2.1	7.9 ± 0.8	6.3 ± 0.7	5.1 ± 1.0	6.1 ± 1.8	4.1 ± 0.9	7.5 ± 1.6
EOS (%)	6.0 ± 0.6	3.0 ± 1.1	4.4 ± 0.6	5.6 ± 1.0	7.5 ± 2.9	6.4 ± 0.7	7.6 ± 2.2	5.7 ± 2.0	7.7 ± 1.9	6.1 ± 0.8
BASO(%)	0.1 ± 0.1	0.0 ± 0.0	0.0 ± 0.0	0.1 ± 0.1	0.1 ± 0.2	0.1 ± 0.1	0.0 ± 0.0	0.0 ± 0.1	0.2 ± 0.1	0.2 ± 0.3

Figure S7. OptiCAR-NK cells exhibit safety in vivo even at elevated administration levels.

(A) Administration of L1 (1.0 or 5.0 x 10^6 cells/mouse) on NSG mice to assess dose-dependent in vivo adverse effects. (B) Changes in body weight and organ weights of liver, lung, heart, spleen, and kidney in male and female mice following treatment with different doses (1.0 or 5.0 x 10^6 cells/mouse) of OptiCAR-NK and its parental NK92 cells. Statistical significance was determined by two-way ANOVA. (C) Results of blood composition analysis in male and female mice following OptiCAR-NK injection.

Supplementary Table 1

Property	Target	Clone #11	Clone #14	Implication	Data source
**Cytotoxicity**	Tumor	- NK92 cell line based CAR: 70-90%<br>- Primary NK based CAR: 80-90%	- NK92 cell line based CAR: 80-90%<br>- Primary NK based CAR: 95-100%	Both clones showed comparable efficacy, robustly eliminating $CD5^+$ tumors	Figures 3B, 4B, 5E Supplementary Fig. 2C
	Normal	- NK92 cell line based CAR: 5-10%<br>- Primary NK based CAR: 20-40%<br>- mRNA assay: lower than 30%	- NK92 cell line based CAR: 15-20<br>- Primary NK based CAR: 40-60%<br>- mRNA assay: lower than 50%	#11 exhibits lower off tumor toxicity than #14, supporting safer therapeutic potential	
**Signaling Strength**	Tumor	- pERK/ERK: 4.67<br>- pAKT/AKT: 1.48	- pERK/ERK: 8.26<br>- pAKT/AKT: 1.21	Clone #14 showed stronger signaling than #11 in both malignant and normal T cells, indicating a higher risk of off-tumor activation.	Supplementary Fig. 3
	Normal	- pERK/ERK: 3.58<br>- pAKT/AKT: 0.78	- pERK/ERK: 4.72<br>- pAKT/AKT: 1.06		
**Binding affinity**	Tumor	- Phage cell binding: 5.5 fold<br>- Phage ELISA: 1.05 O.D.<br>- Conjugation assay: 2-15%	- Phage cell binding: 4.1 fold<br>- Phage ELISA: 2.99 O.D.<br>- Conjugation assay: 7-17%	Both clones exhibited high and specific binding to $CD5^+$ cells, confirming effective antigen recognition	Supplementary Fig. 1
**Cytokine release**	Tumor	-IFN-γ: 9-10pg/ml	-IFN-γ: 9-10pg/ml	Both clones showed comparable cytokine release upon $CD5^+$ target engagement.	Figure 3E

Supplementary Table1. Comparison of Key Properties Between scFv Clones #11 and #14 in CD5 CAR-NK Cells
